# Demographic and migration-related risk factors for low-level smoking in a farm working sample of Latinos (the MICASA study)

**Published:** 2014

**Authors:** Erik J. Rodriquez, Maria T. Stoecklin-Marois, Tamara E. Hennessy-Burt, Daniel J. Tancredi, Marc B. Schenker

**Affiliations:** 1 University of California, Western Center for Agricultural Health and Safety, Center for Tobacco Control Research and Education; 2 University of California, Davis Department of Public Health Sciences, Western Center for Agricultural Health and Safety; 3 UC Davis School of Medicine Department of Pediatrics, Western Center for Agricultural Health and Safety

**Keywords:** Acculturation, Agriculture, Epidemiology, Hispanics/Latinos, Psychosocial, Smoking

## Abstract

Cigarette smoking is the most preventable cause of death in the U.S. Research regarding the phenomenon of low-level smoking, defined as smoking one to five cigarettes per day (CPD) on average, is increasing as its high prevalence is better recognized. The Mexican Immigration to California: Agricultural Safety and Acculturation (MICASA) study is a prospective cohort study of Latino hired farm worker families that assesses respiratory health, including patterns and behaviors of cigarette smoking. The purpose of the present analysis was to establish demographic, migration-related, and psychosocial characteristics and risk factors for low-level smoking. Seven percent of participants were current smokers, 61% of them being low-level smokers. Low-level smokers did not smoke as many days during the past month as those who smoke 6+ CPD (p=0.04). Low-level smokers were more likely than never and former smokers combined not to be married and to experience frequent mental distress. Those who smoke 6+ CPD were also more likely than never and former smokers combined to experience frequent mental distress and to be more acculturated. Low-level smokers have characteristics and risk factors that set them apart from other types of smokers. This increased understanding of low-level smokers can enhance public health education and smoking cessation programs targeted at Latinos.

## 1. Background

The most preventable cause of death in the U.S. is cigarette smoking (Mokdad, Marks, et al., 2004, Mokdad, Marks, et al., 2005). In national and regional surveys Latinos have been found to smoke at a lower rate than non-Latino Whites and non-Latino Blacks. The national estimate of smoking prevalence among adult Latinos is 14.5% compared to 22.1% of non-Latino Whites and 21.3% of non-Latino Blacks (Centers for Disease Control and Prevention, 2010). Rates of smoking among Latinos have been found to differ by sex, ethnicity, and acculturation level. Nineteen percent of Latino men smoke compared to only 9.8% of Latino women (Centers for Disease Control and Prevention, 2010). Among Latino ethnic groups, rates of smoking are highest among Puerto Ricans and lowest among Central Americans (Perez-Stable, Ramirez, et al., 2001). Among Latino women, higher levels of acculturation are associated with increased smoking (Markides, Coreil and Ray, 1987, Marin, Perez-Stable and Marin, 1989, Haynes, Harvey, et al., 1990, Coreil, Ray and Markides, 1991, Samet, Howard, et al., 1992, Palinkas, Pierce, et al., 1993, Cantero, Richardson, et al., 1999, Coonrod, Balcazar, et al., 1999, Sundquist and Winkleby, 1999, Acevedo, 2000). However, associations between acculturation and smoking among men is less consistently reported (Bethel and Schenker, 2005).

More recently, studies have examined the phenomenon of low-level smoking, defined as smoking one to five cigarettes per day (CPD) on average. A daily smoker is someone who reports smoking every day while a non-daily smoker reports smoking only some days. Among adult smokers in the U.S., Latinos are much more likely than non-Latinos to be low-level daily smokers (Trinidad, Perez-Stable, et al., 2009). In California, 70% of Latino smokers are either low-level or non-daily smokers (Zhu, Pulvers, et al., 2007). Further, the number of cigarettes smoked per day is lower for Latinos. Nationally, Latino smokers on average smoke 6.7 CPD compared to 14.9 CPD for non-Latino White smokers and 9.3 CPD for non-Latino Black smokers (Substance Abuse and Mental Health Services Administration, 2006). Smoking among Latino farm worker populations has been found to be predominantly low-level smoking (Gamsky, Schenker, et al., 1992, Garcia, Matheny Dresser and Zerr, 1996).

Research on the prevalence of low-level smoking in the U.S. is limited. Even less data exist on the characteristics, risk factors, and public health significance of low-level smoking (Reitzel, Costello, et al., 2009). The purpose of this study was to characterize low-level smokers and identify demographic, migration-related, and psychosocial risk factors for low-level smoking in a population of Latino farm workers in California.

## 2. Methods

### 2.1 Study Design and Recruitment

The Mexican Immigration to California: Agricultural Safety and Acculturation (MICASA) study is a prospective cohort study conducted among Latino hired farm worker families (Stoecklin-Marois, Hennessy-Burt and Schenker, 2011). Sampling consisted of households residing in the town of Mendota, located in the San Joaquin Valley of California. Mendota was chosen for its large proportion of agricultural workers and Latino immigrants (U.S. Census Bureau). A two-stage stratified area probability sampling design was used. In the first step census blocks were randomly selected from a list of all census blocks in Mendota and enumerators walked door-to-door to map out all dwellings in 62 selected census blocks. In the second step enumerators acquired demographic information about adult individuals residing in each dwelling including age, sex, years lived in Mendota, and involvement in agricultural work. Households that contained at least one hired farm worker were randomly ordered and contacted sequentially for recruitment. Further details regarding the sampling design methods and recruitment of participants have been described previously (Stoecklin-Marois, Hennessy-Burt and Schenker, 2011).

### 2.2 Informed Consent

Prior to obtaining written informed consent, a verbal and written description of the study objectives and procedures were provided to each participant. The study description and written informed consent were provided in Spanish, the primary language of participants. All study procedures were approved by the University of California, Davis Institutional Review Board.

### 2.3 Participant Eligibility

Men and women were eligible to participate in the study if they were 18 to 55 years of age, self-identified as Mexican or Central American, resided in Mendota at the time of the baseline interview, and worked or had a household member who worked in agriculture for at least 45 days in the last year. Eligibility for the present analysis included completion of both the baseline and follow-up interviews.

### 2.4 Rates of Participation

From these households, 803 participants completed the baseline interview and 620 (77.2%) subsequently completed the follow-up interview. For individuals who were not recruited, reported reasons for not participating included distrust, no time or interest, and unwillingness to disclose personal information.

### 2.5 Data Collection and Questionnaire Instruments

The recruitment and baseline interviews of participants were conducted between January 2006 and April 2007. Participant follow-up interviews were conducted between November 2008 and February 2010. Both the baseline and follow-up questionnaires were interviewer-administered in Spanish and most interviews were conducted in the participant's home. Both the baseline and follow-up interviews assessed demographic characteristics, work history, smoking and psychosocial factors. Migration-related factors were assessed only at the baseline interview and frequent mental distress was assessed for the first time at the follow-up interview.

### 2.6 Demographic Characteristics and Migration-Related Factors

Demographic characteristics assessed included participant sex, date of birth, marital status, educational attainment, annual household income, and number of years worked in agriculture. Migration-related factors included country of birth, age at immigration to the U.S., number of years lived in the U.S., and acculturation level. In order to better capture the multi-dimensionality of an individual's level of acculturation, the revised version of the Acculturation Rating Scale for Mexican Americans (ARSMA-II) was used (Cuellar, Arnold and Maldonado, 1995). Two acculturation level categories were established: low and medium or high (medium/high).

### 2.7 Smoking-Related Outcomes and Low-Level Smoking

Questions from the ATS-DLD-78-A were used to assess cigarette smoking (American Thoracic Society). Participants who reported ever smoking at least 100 cigarettes (5 packs) in the follow-up interview were classified as smokers. Those who reported smoking a cigarette within the past 30 days of the follow-up interview were identified as current smokers. Current smokers were then classified as either low-level smokers, defined as individuals smoking one to five CPD on average since they began smoking, or individuals smoking 6+ CPD on average according to the follow-up interview. Both smoking groups included daily and non-daily smokers. Individuals who met the criterion for smoking but had not smoked a cigarette within the past 30 days were categorized as former smokers.

Three participants were excluded from the analysis because their smoking status at the follow-up interview could not be determined. These three participants reported current smoking at the baseline interview but never smoking at the follow-up interview. Three additional participants were missing data for the age that they last smoked cigarettes. These data were imputed by subtracting the number of years since each participant quit smoking cigarettes from that participant's age at the follow-up interview.

### 2.8 Psychosocial and Quality of Life Factors

Among the psychosocial factors assessed in the baseline interview were depressive symptoms, perceived stress, family support, and *nervios*. Depressive symptoms were assessed using a validated screening instrument developed from questions on the Center for Epidemiologic Studies Depression Scale (CES-D) and the Diagnostic Interview Schedule (DIS) from the National Institutes of Mental Health (Burnam, Wells, et al., 1988). Items were coded according to the method by Burnam and colleagues later creating a probability of depressive symptoms. The Perceived Stress Scale questions were rated on a Likert scale and included the following: “how often have you dealt successfully with daily problems and hassles?”, “how often have you coped well with important changes that were taking place in your life?”, “how often have you felt confident about your being able to handle your personal problems?”, “how often have you been able to control your anger in your life?”, “how often have you felt that you were on top of things?”, and “how often did you feel that things were going well?” (Cohen, Kamarck and Mermelstein, 1983).

The assessment of family support was based upon seven items from the Provisions of Social Relations Scale and consisted of the following statements: “no matter what happens, I know that my family will always be there for me should I need them”, “I'm not sure if I can completely rely on my family”, “my family lets me know they think I'm a worthwhile person”, “people in my family have confidence in me”, “people in my family provide me with help in finding solutions to my problems”, “I know my family will always stand by me”, and “I know I can count on my family for financial assistance should I need it” (Turner, Frankel and Levin, 1983). Cronbach's alpha coefficients for perceived stress and family support were 0.80 and 0.88, respectively. Scores for both perceived stress and family support were created by summing responses for each item; with higher scores indicating higher levels of stress and family support. These scores were then dichotomized by using the mean and/or median as a cut-off point. Scores above this established cut-off point were designated as having a “high” level of perceived stress or family support.

A culturally-specific condition known as *nervios* was assessed. *Nervios* has been previously described as a generalized condition of distress that can be expressed with somatic and psychological symptoms (Salgado de Snyder, Diaz-Perez and Ojeda, 2000). Participants were classified as having *nervios* based upon an affirmative response to the question “sometimes in your life, have you ever suffered from *nervios*?”. Fair or poor (fair/poor) self-rated health and frequent mental distress were assessed using the Healthy Days Core Module of the Health-Related Quality of Life instrument from the Centers for Disease Control and Prevention (CDC HRQOL – 4) (Centers for Disease Control and Prevention).

### 2.9 Statistical methods

Univariate analyses were performed on all variables by calculating means, medians, and standard deviations for continuous variables and frequencies and proportions for nominal and ordinal variables. Bivariate analyses examined smoking outcome variables by each predictor of interest. One-way analysis of variance F-tests and chi-square tests for association were used to assess statistical significance by smoking group for continuous and categorical study variables, respectively. Multinomial logistic regression was used to model low-level smoking and smoking 6+ CPD, separately, against the reference category of never and former smoking combined (never/former smoking). Survey data analysis procedures for logistic regression analyses were used to adjust confidence intervals and hypotheses tests for the probability sampling design. All analyses were performed using Statistical Analysis Software, Version 9.2 (SAS Institute Inc., Cary, North Carolina).

## 3. Results

Since patterns of smoking differ between men and women, analyses of demographic characteristics were stratified by sex. Mean age of participants at the follow-up interview was 40.8 years, but men tended to be older than women (p<0.01) (Table 1). The cohort was roughly balanced by sex with 45% men and 55% women. Although 95% of the cohort was married or living with someone at the follow-up interview, a significantly larger proportion of women reported being single, divorced, separated, or widowed (p<0.01). Women were more educated than men; 39% of women versus 29% of men had completed a higher than primary school education (p=0.03). Average annual incomes were low in the population with over three fourths of participants reporting household incomes <$30,000.

One hundred percent of men versus 83% of women ever worked in agriculture (p<0.0001; data not shown). Additionally, men had significantly more work experience in agriculture than women with men reporting an average of 17.7 years compared to 10.5 years for women (p<0.0001).

Sixty-eight percent of participants were born in Mexico and 29% of participants were born in either El Salvador or another Central American country. Overall, the average number of years lived in the U.S. was 15.6 and men had a longer residency than women (18.4 vs. 13.5 years, p<0.0001). Men immigrated to the U.S. 2.5 years earlier, on average, than women. Despite men immigrating earlier and living in the U.S. longer, men and women did not differ significantly by acculturation level. The vast majority of both sexes were classified with a low level of acculturation.

The prevalence of cigarette smoking at follow-up was low; 7% of participants were current smokers and 12% were former smokers (data not shown). Both current smoking and former smoking were more prevalent among men than women (12% versus 4% and 21% versus 4%, respectively; p<0.0001). Sixty-one percent of current smokers compared to 38% of former smokers were low-level smokers (p=0.02). Relationships on smoking behavior among former, low-level, and 6+ CPD smokers showed no differences in the age individuals began smoking or the number of years smoked prior to immigration to the U.S. (Table 2). Participants who started smoking after immigrating to the U.S. were coded as having zero years smoked prior to immigration to the U.S. However, low-level smokers smoked fewer CPD than either former smokers or those who smoke 6+ CPD (p<0.0001). Additionally, the number of years smoking and pack years differed significantly across the groups, with former smokers reporting the shortest number of years smoking and low-level smokers reporting the smallest number of pack years (p<0.0001). Low-level smokers also smoked significantly fewer days in the past month than those who smoke 6+ CPD (p=0.04).

A larger proportion of women were never/former smokers than men (p<0.01) (Table 3). The proportion of never/former smokers who were foreign born was larger than the proportion of low-level smokers who were foreign born, which were both larger than the proportion of those who smoke 6+ CPD who were foreign born (p=0.045).

Multinomial logistic regression models adjusted for age and sex examined associations between demographic and quality of life factors with low-level smoking and smoking 6+ CPD compared to never/former smoking (Table 4). Compared to currently married persons, single, divorced, widowed, or separated participants were relatively more likely to be low-level smokers than never/former smokers (relative rate ratio = 5.04, 95% *CI*: 1.43 – 17.70). Additionally, experiencing frequent mental distress was associated with a greater than threefold higher relative rate of being a low-level smoker (relative rate ratio = 3.47, 95% *CI*: 1.03 – 11.64).

Medium/high acculturated participants were more than six times relatively more likely than low acculturation participants to smoke 6+ CPD (relative rate ratio with respect to never/former smoking prevalence = 6.41, 95% *CI*: 1.23 – 33.39) (Table 4). Similar to the results for low-level smokers, those experiencing frequent mental distress were more than four times relatively more likely to smoke 6+ CPD than to be never/former smokers (relative rate ratio = 4.07, 95% *CI*: 1.31 – 12.69). Compared to others, individuals with more than fifteen years residing in the U.S. were more than two and a half times relatively more likely (with respect to never/former smoking) to be low-level smokers (relative rate ratio = 2.60, 95% *CI*: 1.15 – 5.88) and more than three times relatively more likely of smoking 6+ CPD (relative rate ratio = 3.75, 95% *CI*: 1.12 – 12.57).

## 4. Discussion

The present analysis was one of the first to investigate the characteristics and risk factors of low-level cigarette smoking in a cohort population of Latino farm worker families. These results illustrate that, among Latino farm workers, low-level smokers can be described as having different characteristics than other types of smokers. First, current smokers are more likely to be low-level smokers than smoke 6+ CPD. This is an important finding with the potential to impact public health education and smoking cessation programs. Second, low-level smokers do not smoke as many days during the month as those who smoke 6+ CPD. Third, the number of years a low-level smoker has smoked was lower than that of those who smoke 6+ CPD and higher than that of former smokers. These results are noteworthy because it may be an indication that low-level smokers increase the number of cigarettes they consume over time.

Coinciding with previous research, low-level cigarette smoking is very common among Latino farm workers who currently smoke. A study conducted in Indiana among Latino farm workers found that over 75% of smokers smoked less than 10 CPD (Garcia, Matheny Dresser and Zerr, 1996). In California, the median number of cigarettes smoked per day by male and female farm workers were 5 and 3, respectively (Gamsky, Schenker, et al., 1992). This finding improves our understanding of the smoking behaviors of Latino farm workers who smoke--that current smokers are more likely to be low-level smokers than 6+ CPD smokers, and is useful to designers of public health education and smoking cessation programs.

Statewide, representative data in California has shown that the prevalence of daily low-level smoking among Latinos in the general population is between 16% and 22% (Zhu, Pulvers, et al., 2007). However, among non-daily Latino smokers the prevalence of low-level smoking is between 80% and 85%. In the present analysis, we could not clearly identify daily and non-daily smokers because smokers were not asked whether they smoke “every day” or “some days”.

However, the prevalence of smoking 30 days in the past month among low-level smokers was 54% and among those who smoke 6+ CPD was 83% (p=0.04). This would indicate that low-level smokers in the present analysis are non-daily smokers. Not only is the prevalence of low-level smoking high among Latinos, but Latinos have been found to have increased odds of low-level smoking compared to Blacks or Asian/Pacific Islanders. Using nationally representative data, from the Tobacco Use Supplement of the Current Population Survey, Trinidad and colleagues estimated that Latinos have 4.6 higher odds of being low-level daily smokers compared to Non-Latino Whites (Trinidad, Perez-Stable, et al., 2009).

The characteristics and risk factors of low-level smoking in the Latino population have not been well established in the literature. One study examining low-level smoking attempted to understand the associations of demographic characteristics, tobacco dependence, withdrawal, and cessation with low-level smoking among Latinos from a randomized clinical control trial (Reitzel, Costello, et al., 2009). Research into the characteristics and risk factors of low-level smoking among other ethnic groups is limited to a few studies that examined associations with smoking ≤10 CPD. Among African Americans, smoking ≤10 CPD in young adulthood has been linked to factors in late adolescence such as perceived discrimination, peer smoking, and youth maladaptive characteristics as well as less parental educational attainment and parental smoking (Fagan, Brook, et al., 2009). Among Asian Americans, being a woman, highly educated, not Korean (compared to Chinese), and being a bilingual speaker with high English proficiency compared to being an English-only speaker were factors associated with smoking ≤9 CPD (Tong, Nguyen, et al., 2009).

In the present analysis, being single, divorced, widowed, or separated was found to be strongly associated with being a low-level smoker. Research has found that low-level smokers are more likely not to be married (Hyland, Rezaishiraz, et al., 2005). Additionally, frequent mental distress was associated with a more than threefold higher relative rate of low-level smoking (with respect to never/former smoking). Reasons why marital status and mental distress are associated with low-level smoking have not been well investigated. Being married or living with someone may be protective of social pressures to smoke from other family members, friends, and coworkers (Coreil, Ray and Markides, 1991).

A population-based study using data from the California Tobacco Survey by Zhu and colleagues found that only 36% of low-level smokers at baseline remained low-level smokers 20 months later compared to 82% of 6+ CPD daily smokers. Additionally, they observed that 21% of low-level smokers at baseline increased their cigarette consumption to that of 6+ CPD daily smokers 20 months later (Zhu, Sun, et al., 2003). These findings give some context to the results observed in the present analysis regarding the number of years smoking and smoking group. Low-level smokers were found to smoke for fewer years than those who smoke 6+ CPD. This may be because low-level smokers are more likely to change their cigarette consumption over time.

Conclusions drawn from the present analysis should be interpreted with caution due to the small numbers of smokers in each group. Other limitations due to the sample size of current smokers include the inability to identify characteristics and establish risk factors by sex, Latino ethnicity, or daily/non-daily status. However, strengths of the present study include its use of data from a representative, random sample of farm worker families in California, its effort to describe and characterize low-level smokers using demographic, migration-related, and psychosocial approaches, and its contribution to the understanding of the high prevalence of low-level smoking among Latinos. These findings warrant consideration of smoking behaviors, demographic characteristics, and quality of life factors when targeting current smokers for smoking education and cessation programs; particularly among Latinos and underserved populations such as farm workers. Among the recommendations for future research are to investigate the self-perceptions of low-level smokers as being smokers or non-smokers and to examine the characteristics and risk factors of daily and non-daily low-level smoking separately.

## Figures and Tables

**Table 1:** Description of the study cohort at the follow-up interview (2008-2010).

	Total (n = 620)		Men (n = 277)		Women (n = 343)	
Age, mean ± SD**	40.8 ± 10.2		42.4 ± 10.7		39.5 ± 9.7	
Married/living with someone, % (n)**	95	(586)	97	(270)	92	(316)
Highest grade completed in school, % (n)*						
No school	5	(31)	5	(15)	5	(16)
Primary or less	60	(372)	65	(181)	56	(191)
Greater than primary	35	(215)	29	(81)	39	(134)
Household income, % (n)*						
<$30,000	76	(445)	75	(194)	79	(251)
≥$30,000	24	(138)	26	(67)	22	(71)
Years worked in agriculture, mean ± SD^a^***	14.0 ± 9.7		17.7 ± 10.2		10.5 ± 7.7	
Country of birth, % (n)^a^						
U.S.	3	(20)	2	(7)	4	(13)
Mexico	68	(423)	67	(185)	70	(238)
El Salvador	26	(161)	29	(80)	23	(81)
Other Central American	3	(16)	2	(5)	3	(11)
Age at immigration to U.S., mean ± SD^a^***	23.4 ± 7.7		22.0 ± 6.9		24.5 ± 8.2	
Years lived in the U.S., mean ± SD^a^***	15.6 ± 9.9		18.4 ± 9.9		13.5 ± 9.3	
Low acculturation level, % (n)^a^	97	(581)	97	(265)	96	(316)
Fair/poor self-rated health*	45	(279)	40	(112)	49	(167)

a Data assessed at the baseline interview.

* p<0.05 for the comparison between men and women.

** p<0.01 for the comparison between men and women.

*** p<0.0001 for the comparison between men and women.

**Table 2:** Smoking behavior among former smokers, low-level smokers, and those who smoke 6+ cigarettes per day (CPD).

	Total (n= 114)		Former (n= 68)		Low-level (n= 28)		6+ CPD (n= 18)	
Age began smoking, mean ± SD	18.3 ± 7.2		18.6 ± 7.8		17.8 ± 4.1		18.1 ± 8.7	
Years smoked before immigrating, mean ± SD	6.5 ± 8.3		7.0 ± 7.5		4.2 ± 6.3		8.4 ± 12.4	
Since began smoking…								
Cigarettes smoked per day, mean ± SD***	8.1 ± 6.9		9.0 ± 6.6		3.1 ± 1.5		12.7 ± 8.5	
In the past 30 days…								
Days smoked, mean ± SD*	21.7 ± 12.0		—		18.7 ± 13.0		26.2 ± 8.9	
Years smoking, mean ± SD***	21.3 ± 13.0		16.9 ± 11.0		25.3 ± 11.9		32.4 ± 14.2	
Pack years, mean ± SD***	9.2 ± 12.5		7.7 ± 8.2		3.9 ± 2.8		23.4 ± 22.8	
Lives with someone who smokes, % (n)^a^*	17	(19)	10	(7)	21	(6)	33	(6)
Other tobacco products, % (n)	5	(5)	3	(2)	7	(2)	6	(1)

a Data assessed at the baseline interview.

* p<0.05 for the comparison between all groups.

*** p<0.0001 for the comparison between all groups.

**Table 3:** Demographic and migration-related characteristics of never and former smokers combined, low-level smokers, and those who smoke 6+ cigarettes per day (CPD).

	Total % (n) (n= 620)		Never & former % (n) (n= 574)		Low-level % (n) (n =28)		6+ CPD % (n) (n= 18)	
Sex**								
Male	45	(277)	43	(245)	71	(20)	67	(12)
Female	55	(343)	57	(329)	29	(8)	33	(6)
Foreign birth^a^*	97	(600)	97	(558)	93	(26)	89	(16)
Married/living with someone*	95	(586)	95	(546)	86	(24)	89	(16)
Lived >15 years in the U.S.^a^**	43	(267)	41	(236)	64	(18)	72	(13)
Low acculturation level^a^	97	(581)	97	(540)	93	(26)	88	(15)

a Data assessed at the baseline interview.

* p<0.05 for the comparison between all groups.

** p<0.01 for the comparison between all groups.

**Table 4:** Crude and adjusted odds ratios and 95% confidence intervals (CI) for low-level smoking and for smoking 6+ cigarettes per day (CPD) when compared to never and former smoking combined^a^

	Low-level smoking				Smoking 6+ CPD			
	Crud OR (95% CI)		Adjusted OR (95% CI)^b^		Crude OR (95% CI)		Adjusted OR (95% CI)^b^	
Age: >40	1.24	(0.60 – 2.54)	—	—	**5.36**	**(1.01 – 28.52)**	—	—
Sex: female	**0.30**	**(0.11 – 0.85)**	—	—	**0.37**	**(0.15 – 0.93)**	—	—
Foreign birth	0.37	(0.09 – 1.52)	0.30	(0.08 – 1.14)	**0.23**	**(0.06 – 0.95)**	0.19	(0.03 – 1.07)
Marital status: single/div./wid./sep.	**3.25**	**(1.09 – 9.71)**	**5.04**	**(1.43 – 17.70)**	2.44	(0.51 – 11.70)	3.30	(0.56 – 19.36)
Lived >15 years in the U.S.	**2.60**	**(1.15 – 5.88)**	2.35	(0.79 – 6.98)	**3.75**	**(1.12 – 12.57)**	1.37	(0.23 – 8.14)
Acculturation level: medium/high	2.44	(0.58 – 10.30)	3.15	(0.73 – 13.52)	**4.24**	**(1.15 – 15.55)**	**6.41**	**(1.23 – 33.39)**
Depressive symptoms	1.26	(0.37 – 4.22)	1.42	(0.43 – 4.75)	1.62	(0.53 – 5.03)	1.38	(0.49 – 3.91)
Perceived stress: high	0.99	(0.47 – 2.12)	1.23	(0.56 – 2.69)	0.51	(0.18 – 1.45)	0.55	(0.19 – 1.59)
Nervios	0.83	(0.34 – 2.02)	1.12	(0.46 – 2.72)	2.00	(0.68 – 5.89)	2.53	(0.85 – 7.56)
Family support: high	0.82	(0.44 – 1.55)	0.77	(0.40 – 1.47)	0.64	(0.22 – 1.83)	0.47	(0.16 – 1.42)
Fair/poor self-rated health	0.68	(0.29 – 1.58)	0.70	(0.28 – 1.74)	2.25	(0.67 – 7.53)	1.70	(0.53 – 5.43)
Frequent mental distress	**2.75**	**(1.04 – 7.22)**	**3.47**	**(1.03 – 11.64)**	**4.58**	**(1.50 – 13.96)**	**4.07**	**(1.31 – 12.69)**

a Reference category: never and former smoking combined.

b Adjusted for age and sex.
